# Supplementary material for: Prevalence of Internet Addiction and Gaming Disorders in Southeast Asia: A Meta-Analysis
Source: Int J Environ Res Public Health. 2020 Apr 9;17(7):2582. doi: 10.3390/ijerph17072582 (PMC7177828; doi:10.3390/ijerph17072582)
Supplement: Supplementary file 1 [file ijerph-17-02582-s001.pdf]

Supplementary Materials: Search Strategies

A. MEDLINE Search Strategy

Database: Ovid MEDLINE(R) and Epub Ahead of Print, In-Process & Other Non-Indexed Citations, Daily and Versions(R) <1946 to January 28, 2020>
Final Search: 15
Search Strategy:

----

1 exp epidemiology/ (26334)
2 exp prevalence/ (282151)
3 (epidemiolog$ or prevalanc$).ab,ti,tw. (363600)
4 1 or 2 or 3 (624165)
5 exp Internet/ (76661)
6 exp Video Games/ (5127)
7 (internet or gaming or "digital gaming").ab,ti,tw. (50927)
8 5 or 6 or 7 (109301)
9 exp pathological gambling/ (5350)
10 (addict$ or disorder$ or problematic$ or patholog$ or excessive$ or dependen$ or "addictive behaviour").ab,ti,tw. (3478011)
11 10 or 9 (3480386)
12 exp Southeast Asia/ (92252)
13 (Thai$ or Singapore$ or Malaysia$ or Indonesia$ or Brunei$ or Myanmar$ or Vietnam$ or Cambodia$ or Lao$ or Filipino$ or Philippines or Southeast Asia).ab,ti,tw. (113564)
14 12 or 13 (138668)
15 4 and 8 and 11 and 14 (16)

B. PsycINFO Search Strategy

Search Date: 29th January 2020
Final Search: S16

Search	Query	No. of Results
S1	DE "Epidemiology" OR DE "Birth Rate" OR DE "Comorbidity" OR DE "Demographic Characteristics" OR DE "Health Status" OR DE "Morbidity" OR DE "Population" OR DE "Risk Assessment"	173,115
S2	epidemiolog* or prevalen*	259,168
S3	S1 OR S2	357,999
S4	DE "Internet" OR DE "Blog"	34,947

S5	DE "Digital Gaming" OR DE "Internet Addiction" OR DE "Online Addiction Measures"	4,008
S6	internet or gaming or "digital gaming" or "video games"	67,143
S7	((internet or gaming or "digital gaming" or "video games") AND (S4 OR S5 OR S6)) AND (S4 OR S5 OR S6)	67,143
S8	DE "Addiction" OR DE "Drug Addiction"	21,571
S9	DE "Disorders" OR DE "Disabilities" OR DE "Lesions" OR DE "Adventitious Disorders" OR DE "Atypical Disorders" OR DE "Behavior Disorders" OR DE "Chronic Illness" OR DE "Communication Disorders" OR DE "Congenital Disorders" OR DE "Disorder Attributes" OR DE "Mental Disorders" OR DE "Physical Disorders" OR DE "Sensory Integration Dysfunction" OR DE "Treatment Resistant Disorders	205,660
S10	DE "Pathology" OR DE "Neuropathology" OR DE "Psychopathology" OR DE "Speech Language Pathology"	50,807
S11	addict* or disorder* or problematic* or excessive* or dependent*	1,543,337
S12	(addict* or disorder* or problematic* or excessive* or dependent*) AND (S8 OR S9 OR S10 OR S11)	1,543,337
S13	DE "Southeast Asian Cultural Groups" OR DE "Vietnamese Cultural Groups"	1,588
S14	thai* or singapore* or malaysia* or indonesia* or brunei* or myanmar* or vietnam* or cambodia* or lao* or filipino* or philippines or southeast asia	48,863
S15	(thai* or singapore* or malaysia* or indonesia* or brunei* or myanmar* or vietnam* or cambodia* or lao* or filipino* or philippines or southeast asia) AND (S13 OR S14)	48,863
S16	((thai* or singapore* or malaysia* or indonesia* or brunei* or myanmar* or vietnam* or cambodia* or lao* or filipino* or philippines or southeast asia) AND (S13 OR S14)) AND (S3 AND S7 AND S12 AND S15)	75

C. Web of Science Search Strategy

Search Date: 29th January 2020
Final Search: #5

Search	Query	No. of Results
#1	TS=(epidemiolog$ or prevalen$) Indexes=SCI-EXPANDED, SSCI, A&HCI, CPCI-S, CPCI-SSH, BKCI-S, BKCI-SSH, ESCI Timespan=All years	519,011

#2	TS=(internet or gaming or "digital gaming" or "video games")<br>Indexes=SCI-EXPANDED, SSCI, A&HCI, CPCI-S, CPCI-SSH, BKCI-S, BKCI-SSH, ESCI Timespan=All years	526,209
#3	TS=(addict$ or disorder$ or problematic$ or pathology$ or excessive$ or dependen$ or "addictive behaviour")<br>Indexes=SCI-EXPANDED, SSCI, A&HCI, CPCI-S, CPCI-SSH, BKCI-S, BKCI-SSH, ESCI Timespan=All years	3,998,338
#4	TS=(thai$ or Singapore$ or Malaysia$ or Indonesia$ or brunei$ or Myanmar$ or Vietnam$ or Cambodia$ or lao$ or filipino$ or Philippines or southeast asia)<br>Indexes=SCI-EXPANDED, SSCI, A&HCI, CPCI-S, CPCI-SSH, BKCI-S, BKCI-SSH, ESCI Timespan=All years	310,583
#5	#4 AND #3 AND #2 AND #1<br>Indexes=SCI-EXPANDED, SSCI, A&HCI, CPCI-S, CPCI-SSH, BKCI-S, BKCI-SSH, ESCI Timespan=All years	10

D. Embase Search Strategy

Database: Embase <1996 to 2020 Week 04>
Final Search: 16
Search Strategy:
----
1 exp epidemiology/ (2973362)
2 exp prevalence/ (694404)
3 (epidemiolog$ or prevalanc$).ab,ti,tw. (385787)
4 1 or 2 or 3 (3161997)
5 exp Internet/ (108421)
6 exp game addiction/ (922)
7 (internet or gaming or "video games" or "digital gaming").ab,ti,tw. (70612)
8 5 or 6 or 7 (133335)
9 exp addiction/ (217750)
10 exp pathological gambling/ (5698)
11 (addict$ or disorder$ or problematic$ or patholog$ or excessive$ or dependen$ or "addictive behaviour").ab,ti,tw. (3695694)
12 9 or 10 or 11 (3789203)
13 exp Southeast Asia/ (104611)
14 (Thai$ or Singapore$ or Malaysia$ or Indonesia$ or Brunei$ or Myanmar$ or Vietnam$ or Cambodia$ or Lao$ or Filippino$ or Philippines or Southeast Asia).ab,ti,tw. (131172)

15 13 or 14 (151691)
16 4 and 8 and 12 and 15 (77)

E. Cochrane Central Search Strategy

Search Date: 30th January 2020
Final Search: #16

Search	Query	No. of Results
#1	MeSH descriptor: [Epidemiology] explode all trees	47
#2	MeSH descriptor: [Prevalence] explode all trees	4,850
#3	(epidemiology or prevalenc*):ti,ab,kw	80,606
#4	#1 or #2 or #3	80,609
#5	MeSH descriptor: [Internet] explode all trees	3,727
#6	MeSH descriptor: [Video Games] explode all trees	622
#7	(internet or gaming or "video games" or "digital gaming"):ti,ab,kw	11,061
#8	#5 or #6 or #7	11,142
#9	MeSH descriptor: [Behavior, Addictive] explode all trees	553
#10	((addict* or disorder* or problematic* or patholog* or excessive* or dependen* or "addictive behaviour")):ti,ab,kw	275,748
#11	MeSH descriptor: [Pathology] explode all trees	56
#12	#9 or #11 or #10	275,749
#13	((Thai* or Singapore* or Malaysia* or Indonesia* or Brunei* or Myanmar* or Vietnam* or Cambodia* or Lao* or Filippino* or Philippines or Southeast Asia)):ti,ab,kw	9,289
#14	MeSH descriptor: [Asia, Southeastern] explode all trees	2,486
#15	#13 or #14	9,309
#16	#4 and #8 and #12 and #15	6

F. PubMed Search Strategy

Search Date: 29th January 2020
Final Search: #5

Search	Query	No. of Results
#1	Search (epidemiology) OR prevalence	2,683,955
#2	Search (Internet) OR gaming	111,422

#3	(((((addiction) OR disorder) OR problematic) OR pathological) OR excessive) OR dependent	8,670,725
#4	Search Southeast Asia	99,944
#5	#1 AND #2 AND #3 AND #4	73
